# Imaging Cyclic AMP Changes in Pancreatic Islets of Transgenic Reporter Mice

**DOI:** 10.1371/journal.pone.0002127

**Published:** 2008-05-07

**Authors:** Joung Woul Kim, Craig D. Roberts, Stephanie A. Berg, Alejandro Caicedo, Stephen D. Roper, Nirupa Chaudhari

**Affiliations:** 1 Department of Physiology and Biophysics, University of Miami School of Medicine, Miami, Florida, United States of America; 2 Program in Neurosciences, University of Miami School of Medicine, Miami, Florida, United States of America; 3 Diabetes Research Institute, University of Miami School of Medicine, Miami, Florida, United States of America; Ordway Research Institute, United States of America

## Abstract

Cyclic AMP (cAMP) and Ca^2+^ are two ubiquitous second messengers in transduction pathways downstream of receptors for hormones, neurotransmitters and local signals. The availability of fluorescent Ca^2+^ reporter dyes that are easily introduced into cells and tissues has facilitated analysis of the dynamics and spatial patterns for Ca^2+^ signaling pathways. A similar dissection of the role of cAMP has lagged because indicator dyes do not exist. Genetically encoded reporters for cAMP are available but they must be introduced by transient transfection in cell culture, which limits their utility. We report here that we have produced a strain of transgenic mice in which an enhanced cAMP reporter is integrated in the genome and can be expressed in any targeted tissue and with tetracycline induction. We have expressed the cAMP reporter in β-cells of pancreatic islets and conducted an analysis of intracellular cAMP levels in relation to glucose stimulation, Ca^2+^ levels, and membrane depolarization. Pancreatic function in transgenic mice was normal. In induced transgenic islets, glucose evoked an increase in cAMP in β-cells in a dose-dependent manner. The cAMP response is independent of (in fact, precedes) the Ca^2+^ influx that results from glucose stimulation of islets. Glucose-evoked cAMP responses are synchronous in cells throughout the islet and occur in 2 phases suggestive of the time course of insulin secretion. Insofar as cAMP in islets is known to potentiate insulin secretion, the novel transgenic mouse model will for the first time permit detailed analyses of cAMP signals in β-cells within islets, i.e. in their native physiological context. Reporter expression in other tissues (such as the heart) where cAMP plays a critical regulatory role, will permit novel biomedical approaches.

## Introduction

Although cyclic adenosine 3′-5′-monophosphate (cAMP) has been recognized as an intracellular second messenger for decades, a detailed understanding of its regulation, compartmentalization, range of subcellular targets, and interactions with other second messenger systems has been difficult to reach, especially in complex tissues. This contrasts with analyses on another ubiquitous second messenger, Ca^2+^, in large part due to the availability of indicator dyes that are readily introduced into cells and tissues. An early microinjectable reporter for cellular cAMP was constructed by labeling Protein Kinase A subunits with fluors for Fluorescence Resonance Energy Transfer (FRET) [1]. Elevated cAMP resulted in dissociation of the subunits, and a resulting graded loss of FRET signal. Genetically encoded reporters have been produced using the cAMP-binding domains of Protein Kinase A [2], [3] or Epac [4], fused to spectrum-shifted variants of Green Fluorescent Protein (GFP). Although such chimeric proteins are effective reporters of cAMP concentration and dynamics, the requirement for transfection limits their use in complex tissues and *in vivo*. Unlike transfected cell lines, tissues from transgenic animals endogenously expressing functional reporters offer the advantage of examining tissues with interacting cell types *ex vivo* or *in vivo*.

In the last few years, functional reporters for calcium [5], [6] and synaptic vesicle release [7] were incorporated into transgenic mice. A reporter for cAMP also was transgenically engineered into mice [8]. However, because cAMP reporters sequester cAMP, chronic changes in cAMP levels might alter tissue growth, development or homeostasis. Thus, we opted to produce transgenic mice in which genes for the cAMP reporter protein are silent until induced by a reverse tetracycline transactivator (rtTA) protein in combination with the tetracycline analog, doxycycline (dox) [9], [10]. This strategy avoids altering the physiological state of transgenic tissues and is readily adapted to target any cell- or tissue-type of interest.

## Results

### Production of transgenic mice with an enhanced cAMP reporter

**Figure 1:**
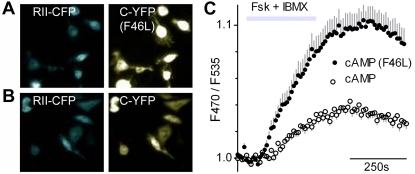
cAMP reporter, enhanced by a mutation in YFP. A, B. CHO cells, stably expressing rtTA, were cotransfected with RII-CFP and either the mutated (F46L, top) or original (lower) C-YFP. Note similar CFP fluorescence (left) but enhanced YFP fluorescence (right) for the F46L mutant. C. Transfected cells from A. and B., functionally imaged for FRET and stimulated with10 µM Fsk+100 µM IBMX to elevate cAMP levels. The F46L mutant cAMP reporter (•) yields a larger peak FRET signal (F470/F535) than the original reporter (○) (mean±s.e.m., n = 18 cells).

The original genetically encoded cAMP reporter [3] consists of two chimeric proteins, Yellow Fluorescent Protein fused to the Protein Kinase A (PKA) Catalytic subunit (C-YFP) and Cyan Fluorescent Protein fused to the PKA Regulatory II subunit (RII-CFP). First, we enhanced the efficiency of this reporter by introducing a single mutation in YFP (phenylalanine to leucine at amino acid 46). This F46L variant of YFP is reported to mature faster, fluoresce brighter, and was predicted to be a more efficient acceptor for FRET [11]. Indeed, we found that cells transfected with F46L cAMP reporter (Figure 1A) produced an enhanced FRET signal (*i.e.* emission at 535 nm following excitation at 430 nm) compared with the original reporter (Figure 1B,C).

**Figure 2:**
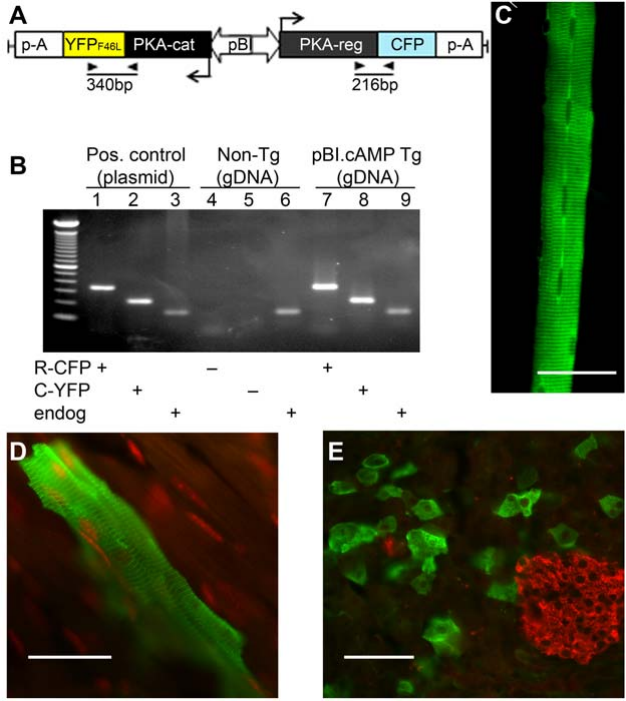
Enhanced cAMP reporter, expressed in tissues of double transgenic CMV-rtTA/pBI-cAMP mice. A. Transgenic construct in pBI vector, with C-YFP and RII-CFP in opposing orientations around a bi-directional tetracycline-inducible promoter. Genotyping primers (▸,◂) and the resulting PCR products are indicated. B. Example of genotyping on genomic DNA (gDNA) from a mouse lacking (non-Tg) or possessing the integrated transgene (pBI-cAMP Tg). PCRs with each template tested for RII-CFP (lanes 1, 4, 7), C-YFP (lanes 2, 5, 8) and an endogenous gene, PLCβ2 (lanes 3, 6, 9). C–E. Tissues from double transgenic CMV-rtTA/pBI-cAMP mice were immunostained with anti-GFP (green) to visualize the reporter in skeletal myofibers (C), cardiac myocytes (D) and pancreas (E). In the pancreas, only acinar cells express the reporter, while islets of Langerhans (immunostained with anti-insulin, red) do not. In D., nuclei are counterstained red with TO-PRO-3. Scale bars, 50 µm.

Next, we produced transgenic mice (Supporting Table S1) in which the enhanced cAMP reporter protein genes are silent until induced by the presence the transactivator, rtTA, and dox [10] (Figure 2A,B and Supporting Figure S1). We termed these transgenic mice “pBI-cAMP”. To reveal expression of the fluorescent reporter, we crossed pBI-cAMP mice separately with two transgenic strains of rtTA transactivator mice obtained from Jackson Laboratories: (1) CMV-rtTA, with a broadly expressed cytomegalovirus promoter and (2) Ins2-rtTA, with a pancreatic β-cell specific promoter.

In CMV-rtTA/pBI-cAMP double transgenic mice, dox induction resulted in expression of RII-CFP and C-YFP in several tissues, including skeletal (Figure 2C) and cardiac (Figure 2D) muscle. In both types of striated muscle, fluorescence was aligned with sarcomeres. We attribute this to the association of expressed RII-CFP subunit with A-Kinase Anchoring Protein (AKAP) which is known to be attached to transverse tubules [12]. In the pancreas of CMV-rtTA/pBI-cAMP mice, fluorescence was present only in acinar cells (Figure 2E). In all three tissues, only a subset of cells of any given type expressed the reporter proteins. This was likely due to the CMV promoter expressing rtTA inefficiently in the CMV-rtTA mice. We also observed reporter expression in the choroid plexus and lung (not shown). No fluorescence was detected in the absence of dox-induction (not shown). We did not characterize these CMV-rtTA/pBI-cAMP mice further.

### Targeted expression of cAMP reporter in pancreatic islets

**Figure 3:**
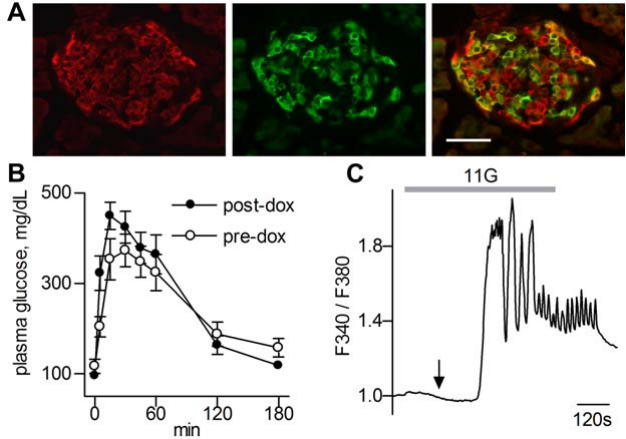
Pancreatic islets function normally in double transgenic Ins2-rtTA/pBI-cAMP mice induced to express the cAMP reporter in pancreatic islet β-cells. A. Cryosections of a pancreas, immunostained with anti-insulin to reveal islets of Langerhans (red) and anti-GFP (green). The overlay (right) shows that only β-cells express the transgenic cAMP reporter. We detected no gross changes in islet histology in transgenic mice. B. Reporter expression does not interfere with glucose homeostasis. Double transgenic mice, subjected to a Glucose Tolerance Test before (○), and 1 week after (•) induction of the cAMP reporter showed similar rise and fall in plasma glucose (mean±s.e.m.; n = 5 mice). C. β-cells from Ins2-rtTA/pBI-cAMP mice show normal glucose-stimulated Δ[Ca^2+^]_i_ (imaged with Fura-2). Glucose was elevated from 3 mM (basal) to 11 mM (grey bar, 11G). Intracellular [Ca^2+^] decreased transiently (arrow), then rapidly increased with a series of oscillations that continued for several minutes after glucose returned to the basal concentration. Similar responses were obtained in islets from wild-type mice (not shown).

In Ins2-rtTA/pBI-cAMP mice, reporter expression was dependent on dox and was limited to β-cells in pancreatic islets of Langerhans (Figure 3A). We noted that not all β-cells expressed the reporter. Further, there was no obvious correlation between the intensity of insulin immunoreactivity and of reporter expression. The mosaic pattern of reporter expression may result simply from variable expression of the rtTA transactivator from the transgenic Ins2 promoter. High levels of rtTA expression are known to be essential for dox-dependent expression [13]. We detected no abnormalities in gross structure or histology of the induced, transgenic pancreas. We then tested whether expression of the cAMP reporter in β-cells interfered with normal pancreatic function. Double transgenic Ins2-rtTA/pBI-cAMP mice were subjected to a glucose tolerance test (GTT) before, and 1 week after, dox-induction. The transient rise of blood glucose and return to resting levels were indistinguishable before and after dox (Figure 3B), and resembled those in normal mice. Thus, cAMP reporter expression for a week did not grossly affect the ability of transgenic mice to exhibit glucose sensing or insulin secretion.

To assess the glucose sensitivity of β-cells more directly, we isolated and cultured islets from dox-induced Ins2-rtTA/pBI-cAMP mice, loaded the islets with Fura-2, and measured glucose-stimulated changes in intracellular Ca^2+^. Isolated islets responded to glucose with an initial slight decrease in [Ca^2+^]_i_, followed by a sharp increase, and subsequent oscillations (Figure 3C). Such Ca^2+^ oscillations are typical of healthy, freshly isolated islets. Further, by measuring Ca^2+^ signals in Regions of Interest (ROIs) in different areas of individual islets, we confirmed that rapid Ca^2+^ oscillations were synchronized across the surface of induced transgenic islets, as widely reported for wild type islets [14]–[16]. These findings suggested that there was no gross alteration in the physiology of β-cells from pBI-cAMP double transgenic mice expressing the cAMP reporter.

### Glucose-evoked cAMP signals in pancreatic β-cells

**Figure 4:**
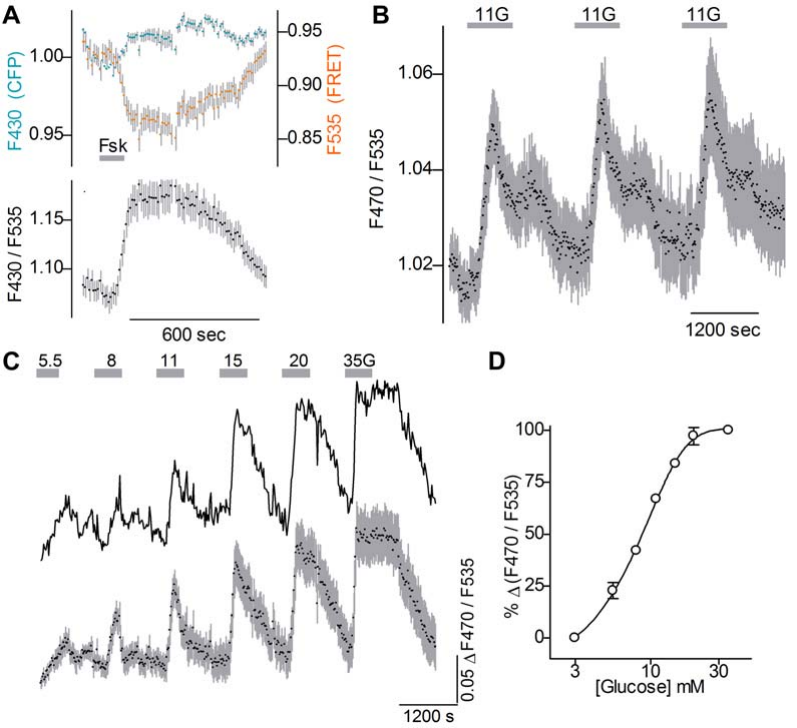
Glucose stimulation results in dynamic and dose-dependent changes of cAMP concentration in β-cells. Islets were harvested from double transgenic Ins2-rtTA/pBI-cAMP mice after induction. A. When cAMP was elevated (grey bar, 10 µM Forskolin) in islets excited at 430 nm, FRET emission (upper, 535 nm, orange symbols) dropped while CFP fluorescence (upper, 470 nm emission, cyan symbols) increased slightly. The ratio of these emission (lower, F470/F535; black symbols) is a monitor of changing cAMP concentration. Example shown is mean±s.e.m. (n = 14 cells in different regions of 1 islet). B. Repeated stimulation with 11 mM glucose (grey bars, 11G) produced consistent cAMP responses in β-cells (mean±s.e.m.; n = 6 cells). C. Stimulating islets with increasing concentrations of glucose (5.5 to 35 mM, grey bars) evoked increasing cAMP responses in β-cells. Upper trace is ratio measurements across a single islet, lower trace is mean±s.e.m. for 4 islets. D. The concentration-response function (mean±s.e.m., n = 4 islets) for cAMP, with EC50 = 9 mM glucose, corresponds well with glucose-stimulated insulin release in isolated islets [17], [18].

We next proceeded to determine whether the reporter monitored intracellular cAMP in Ins2-rtTA/pBI-cAMP mice. Pancreatic islets from dox-induced double transgenic mice yielded a FRET signal when excited at 430 nm, and more importantly, showed a change in FRET intensity when intracellular cAMP was elevated experimentally. Perfusing islets with forskolin (10 µM) plus 3-Isobutyl-1-methylxanthine (IBMX, 100 µM) decreased emission from the yellow fluorophore with no change or a slight increase in emission of the cyan flurophore (colored traces in Figure 4A). Forskolin and IBMX separately also elicited similar changes of FRET intensity, consistent with the interpretation that changes of intracellular [cAMP] generate in the emission ratio (F470/F535, lower trace Figure 4A) [3]. We ruled out that the FRET signal was artifactually generated by other likely metabolic events such as changes of intracellular pH or NADH concentration (Supporting Figure S2).

Having established that a FRET-based signal monitors [cAMP]_i_ we tested responses to glucose. Starting from a resting level of 3 mM glucose, repeated brief applications of 11 mM glucose elicited reproducible cAMP responses with little potentiation or attenuation in successive trials (Figure 4B). Next, we measured the concentration-response relationship for glucose-evoked cAMP increases (Figure 4C,D). The EC_50_ calculated from these data was 9 mM, consistent with steady-state measures of cAMP in glucose-stimulated islets [17], [18].

**Figure 5 pone-0002127-g005:**
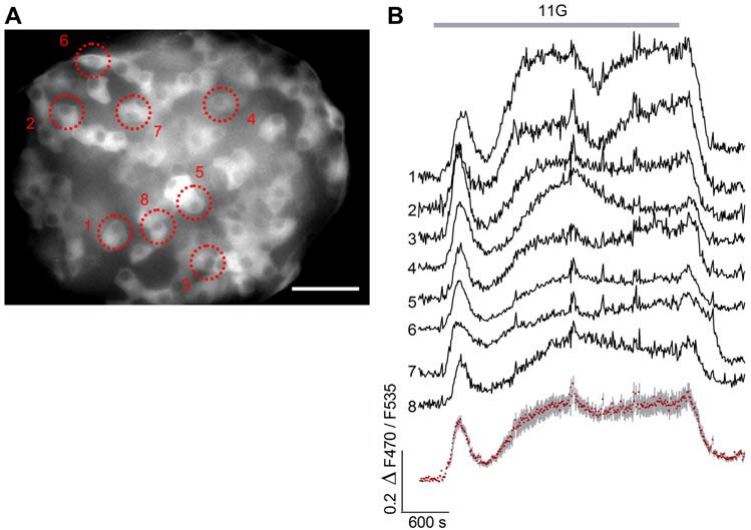
Prolonged stimulation with glucose results in a biphasic pattern of cAMP accumulation in β-cells. A. A living islet from an induced Ins2-rtTA/pBI-cAMP mouse, viewed for YFP fluorescence. Dotted circles are ROIs analyzed functionally, and correspond to numbered traces in B. Scale bar, 20 µm. B. Prolonged glucose stimulation of this islet (grey bar, 11G) resulted in a nearly synchronous, biphasic elevation of intracellular cAMP in β-cells across the surface of the islet (black traces correspond to numbered ROIs shown in A; red symbols are mean±s.e.m. for the 8 ROIs).

We also subjected islets to prolonged elevations of glucose. Extended stimulation with 11 mM glucose elicited a biphasic increase in cAMP: an initial peak was followed by a second, slowly developing plateau (Figure 5B). The cellular resolution (see Figure 5A) afforded by the transgenically expressed cAMP reporter revealed that glucose-evoked Δ[cAMP] was nearly synchronous across the surface of the islet. (To ensure accurate measurements, we imaged only cells on the islet surface for Figure 5). This synchrony is evident in the individual traces in Figure 5B, and the small s.e.m. of the averaged response (colored trace at bottom, Figure 5B). Interestingly, the biphasic kinetics of the cAMP response to prolonged glucose stimulation resembles the commonly observed biphasic secretion of insulin, including in mice [19], [20].

### Glucose-stimulated cAMP accumulation does not require Ca^2+^ elevation

**Figure 6:**
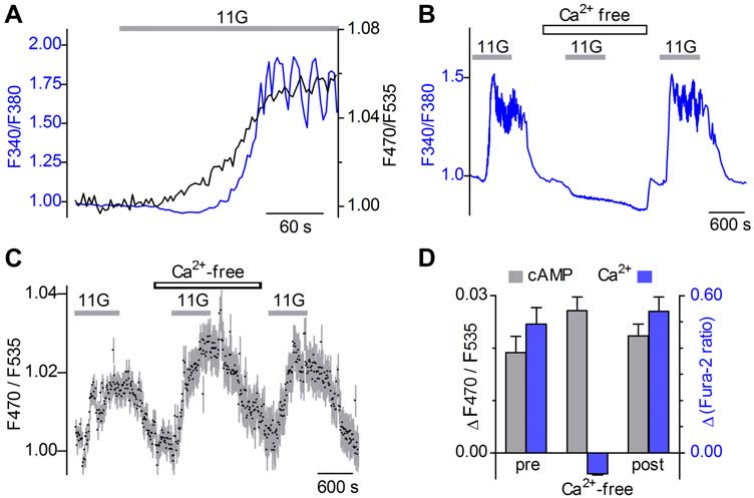
Glucose-stimulated cAMP does not require Ca^2+^ elevation. A. Islets expressing cAMP reporter were loaded with Fura-2 to measure Ca^2+^ and cAMP concurrently. In response to glucose (11G, grey bar), the increase in cAMP (black trace) precedes Ca^2+^ elevation and oscillations (blue trace). B–D. Glucose-stimulated cAMP in β-cells is independent of [Ca^2+^]_i_. B. Glucose–evoked Ca^2+^ oscillations are completely eliminated in the absence of extracellular Ca^2+^, as widely reported for β-cells. Trace shows Fura 2 responses (F340/F380) to 11 mM glucose (grey bars, 11G) before, during and after the depletion of extracellular Ca^2+^. C. In contrast, glucose-evoked cAMP responses persist when extracellular Ca^2+^ is removed. D. Mean responses from Ca^2+^ - and cAMP imaging to 11 mM glucose before, during, and after Ca^2+^ is removed from bath (±s.e.m., n = 6 islets each). Ca^2+^- and cAMP-imaging were conducted independently to prevent any spectral overlap.

We concurrently imaged changes in cAMP and Ca^2+^ by loading islets from transgenic mice with the calcium indicator dye Fura 2, and measuring ΔF470/F535 (for cAMP) and ΔF340/F380 (for Ca^2+^). Our results showed that glucose-stimulated increases in cAMP preceded Ca^2+^ increases. Further, there was no obvious parallel between Ca^2+^ oscillations and cAMP signals (Figure 6A) even though our sampling rate would have detected these. To eliminate the possibility of spectral overlap between the cAMP reporter and Fura-2, we also conducted independent Ca^2+^ and cAMP imaging. In Fura-2-loaded transgenic islets, we confirmed that the glucose-stimulated Ca^2+^ signal disappeared when Ca^2+^ was removed from the bathing medium (Figure 6B). Most or all the glucose-induced elevation of Ca^2+^ is known to occur via influx, not release from stores. In contrast, we found that glucose-evoked cAMP elevation was unaffected by removing Ca^2+^ from the bath (Figure 6C,D). Taken together, these data indicate that glucose-stimulated Ca^2+^ elevation and increases of cAMP arise from distinct cellular mechanisms in β-cells.

Membrane depolarization underlies glucose-stimulated Ca^2+^ influx and insulin secretion in β-cells. To test whether depolarization influences cAMP accumulation, we stimulated islets with 50 mM KCl while imaging cAMP. Whereas KCl elicited reliable and robust Ca^2+^ influx as expected, it did not evoke cAMP responses (data not shown; n = 6 islets in 3 experiments).

### Nuclear translocation of PKA

**Figure 7:**
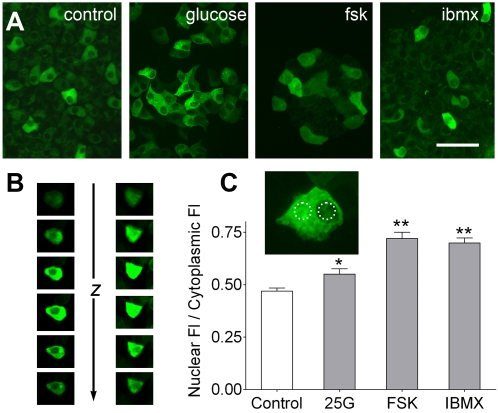
Elevation of cAMP causes PKA catalytic subunit translocation to the nucleus in β-cells. A. Islets from induced Ins2-rtTA/pBI-cAMP mice were incubated for 30 min in control media or with added glucose (25 mM), forskolin (10 µM) or IBMX (100 µM). Nuclear C-YFP fluorescence is visible after prolonged elevation of cAMP (especially with fsk) in contrast to cytoplasmic localization in control islets. Scale bar, 20 µm. B. Z-stacks of confocal images to illustrate cytoplasmic C-YFP (*i.e.* with dark nucleus, left) and nuclear translocated (*i.e.* with bright nucleus, right) following IBMX for 30 min. C. Fluorescence intensity was quantified in Regions Of Interest (dotted circles in inset) over the nucleus and cytoplasm of cells treated as in A. The ratio of nuclear to cytoplasmic fluorescence was significantly higher relative to control when cAMP levels were elevated (* p≤0.05; ** p≤0.01; Dunnett's multiple comparisons test; n = 24–47 cells in 3 experiments for each treatment).

In many tissues, prolonged elevation (10's of min) of cAMP levels causes the dissociated catalytic subunit of PKA to translocate from the cytoplasmic to nuclear compartment where it phosphorylates and activates targets such as CREB, the cAMP Response Element Binding protein [21], [22]. To examine if the YFP-tagged PKA catalytic subunit of the transgenically expressed reporter would exhibit this behavior, we elevated cAMP in islets by incubating them in 25 mM glucose, 10 µM Fsk or 100 µM IBMX, all for 30 min. Each of these treatments resulted in a significant increase of YFP-fluorescence within the nucleus (Figure 7A,B). The effect was more pronounced with Fsk and IBMX, relative to glucose (Figure 7C), in keeping with the higher apparent cAMP accumulation observed in FRET studies using these treatments. The GFP moiety fused to the PKA catalytic subunit did not prevent nuclear translocation in our transgenic model, in contrast to the apparent failure with a different chimeric catalytic subunit tested in transfected β-cells [23].

## Discussion

Here, we have described a new transgenic mouse model that will allow detailed investigations of cAMP signaling in a native, physiological context. A reporter for cAMP has been incorporated into the genome of mice, under a tightly regulated promoter that permits expression in any cell type of interest, and only upon activation with doxycycline. We have expressed the cAMP reporter in β-cells of pancreatic islets and recorded dose-dependent, glucose-stimulated changes in cAMP. The cAMP response appears to be independent of Ca^2+^ elevation and is synchronized throughout the islet. Interestingly, the cAMP signal occurs in an early transient phase and a later plateau phase, which parallels the time course of insulin secretion [19], [20], [24]. Insofar as cAMP in islets is known to potentiate insulin secretion [25]–[27], the novel transgenic mouse model will for the first time permit detailed analyses of second messenger signaling in β-cells *in situ*. Reporter expression in other cells (such as cardiac myocytes) where cAMP also plays a critical regulatory role, will similarly permit detailed analyses.

In response to glucose, β-cells in islets exhibit highly synchronized responses of metabolism, membrane voltage and [Ca^2+^]_i_ which may in turn underlie the finely regulated kinetics of insulin secretion [14]–[16]. We show here that glucose-evoked cAMP signals in β-cells of intact islets also exhibit such synchrony and approximately mirror the kinetics of insulin secretion. We have not explored whether the synchronization of cAMP dynamics is achieved through regulatory circuits involving non-β cells within the islet, or reflects the kinetics of metabolic processes within β cells. The transgenic system we report is ideal for such analyses.

Biphasic insulin secretion reflects an immediate vesicular release followed by a slower mobilization of reserve insulin granules [24], [28]. Recent evidence suggests separate second messenger pathways may underlie these distinct phases [29]. Although cAMP clearly potentiates insulin secretion, the mechanism of this interaction is not understood. cAMP may stimulate vesicular mobilization [26] or may facilitate exocytosis via Epac and PKA pathways [25], [27], [30], or both. Another possible role for glucose-stimulated cAMP is its gating action on Hyperpolarization-activated cyclic nucleotide-gated (HCN) channels, recently identified in islets and in the MIN6 line of β-cells [31]. By regulating membrane voltage via HCN channels, cAMP may help shape the time course and duration of glucose-evoked bursts of action potentials [32]. The transgenic mice that we describe present an opportunity to dissect these processes with spatial and temporal resolution.

Our results, using intact mouse islets, differ from a previous report on a clonal line of β-cells, MIN6 transfected with a cAMP reporter [33]. In that study, glucose-dependent cAMP elevation occurred only after calcium elevation, and large cAMP oscillations (at ≈0.5/min) occurred in synchrony with Ca^2+^ fluctuations. Oscillations of cAMP in transfected clonal β-cell lines were also noted in response to peptide hormones [23]. In contrast, oscillations of cAMP were not a prominent feature in our intact transgenic islets (Figures 4–6). The discrepancy may reflect functional cooperation between adjacent β- and non-β cells, which would be present in our intact islet preparation, but would be lacking in clonal β-cell lines [34].

Based on measurements from islet homogenates and β-cell lines, it has long been known that glucose stimulates cAMP accumulation. Yet, it has not been possible to study the mechanism of cAMP synthesis nore the downstream effects of cAMP within the complex assemblage of cells in intact islets. The binary transgenic mouse we report here presents an opportunity for such measurements. Because many transgenic strains of mice now exist, expressing rtTA in various tissues, the system can also be readily adapted for detailed investigations of cAMP dynamics in other clinically relevant cells including cardiac myocytes and neurons.

## Materials and Methods

### pBI-cAMP and other transgenic mice

The R-CFP and C-YFP subunits of the PKA-based cAMP reporter [3], in pcDNA3, were a generous gift from Dr. M. Zaccolo (Venetian Institute of Molecular Medicine, Padova, Italy). The C-YFP cDNA was released with NotI and XbaI (1818 bp) and cloned into pBI vector (BD Biosciences). Next, R-CFP cDNA was released with NheI and XbaI (2044 bp) and ligated into the opposite cloning site of the vector. A point mutation at amino acid #46 of YFP was introduced by PCR to yield the pBI-cAMP_F46L_ construct (Figure 2A). This plasmid was linearized with AatII and AseI to remove vector sequences and purified. The DNA was injected into fertilized eggs of C57BL/6J×SJL/J mice, and eggs were transfered into foster mothers, all at the University of Miami Transgenic Facility. Founder mice were bred with C57BL/6J mice to establish “pBI-cAMP” transgenic lines expressing the F46L-variant of the original cAMP reporter. We tested each of 7 transgenic lines by culturing fibroblasts from the tail tissue of young mice, and transiently transfecting them with pTet-ON (BD Biosciences). After 2 days, dox (2 µg/ml) was added to the culture for 24 hours and YFP fluorescence was assessed to identify lines in which the transgenes were expressed in a dox- and rtTA-dependent fashion.

Two transactivator lines of mice were purchased from Jackson Laboratory (Bar Harbor, ME): Tg(rtTAhCMV)4Bjd/J (#003273) and NOD.Cg-Tg(Ins2-rtTA)2Doi/DoiJ (#004602). We refer to these strains as CMV-rtTA and Ins2-rtTA respectively. Each strain was separately mated with pBI-cAMP mice and the progeny were interbred to homozygosity for rtTA or for both rtTA and the reporter transgenes. The resulting strains, CMV-rtTA/pBIcAMP or Ins2-rtTA/pBIcAMP mice, are here refered to as “double transgenic mice”.

### Doxycycline (dox) induction

Expression of the pBI-cAMP transgenes was induced by injecting dox (Sigma Chemicals; 100 mg/Kg body weight) intraperitoneally, 4 and 2 days prior to euthanasia. Lower doses or shorter duration of induction appeared to produce lower levels of reporter expression. We also noted that homozygosity of the rtTA allele seemed to be important for high levels of reporter expression sufficient for functional imaging.

### Genotyping and immunohistochemistry

All experimental procedures followed NIH Guidelines for the Care and Use of Animals and were approved by the University of Miami Animal Care and Use Committee. Tail tissue was genotyped as previously described [35] using the following primer pairs: tgccatgtgagcctagcctaag and gcaatagaacagggttgagcaaag for the endogenous PLCβ2 gene; gccgccattattacgacaag and cctcgatggtagacccgtaa for rtTA; atggatgtgcaagcatttga and gtggtgcagatgaacttcag for R-CFP; caatgagaagtgtggcaagg and gtggtgcagatgaacttcag for C-YFP. A common reverse primer in the GFP sequence was used for both R-CFP and C-YFP. All PCRs were carried out for 32 cycles with annealing at 58°C.

Immunohistochemistry was carried out on perfusion-fixed (4% paraformaldehyde) tissues, essentially as decribed previously [35] and using the following antibodies: chicken anti-GFP at 1∶2000 (GFP-1020, Aves Labs, Tigard, OR); guinea pig pre-diluted anti-insulin (AR029-5R, BioGenex, San Ramon, CA); Alexa 488-goat anti-chicken IgG at 1∶2000 (A11039, Invitrogen); Alexa 594-goat anti-guinea pig IgG at 1∶1000 (A11076, Invitrogen). In some instances, nuclei were counterstained with 1 µM TO-PRO-3 (Invitrogen) for 15 min.

### Isolating and culturing pancreatic islets

For functional studies, we used islets from adult (5–14 weeks) male and female Ins2-rtTA/pBI-cAMP mice, dox-induced as above. After euthanasia, collagenase P (2.0–2.5 ml; Roche Diagnostics, Indianapolis, IN) was injected with a 30 ga. needle as a 1 mg/ml solution in Hank's balanced salt solution (HBSS) through the bile duct into the pancreas. The filled pancreas was dissected free, incubated at 37°C for 7–10 min, then triturated in Washing Buffer (HBSS supplemented with 10 mM Hepes and 5% bovine serum albumin) to release individual islets. Such islets were washed 3 times in Washing Buffer at 4°C. Islets were then collected with glass pipettes, and cultured on Celltak-coated coverslips, in Opti-MEM (Invitrogen) supplemented with 10% fetal bovine serum and 2 µg/ml dox.

### cAMP and Ca^2+^ imaging

Islets from Ins2-rtTA/pBI-cAMP mice were isolated and maintained in culture for up to 7 days for functional studies. Coverslips with islets were placed in a recording chamber (Warner Instruments, Hamden, CT) in buffer containing 120 mM NaCl, 4.8 mM KCl, 2.5 mM CaCl_2_, 1.2 mM MgCl_2_, 3 mM glucose, 25 mM NaHCO_3_, 1% BSA and 10 mM HEPES, pH 7.4 [32]. Stimuli were applied by a gravity fed perfusion system at a rate of ∼1 ml/min which produced a full exchange of the bath in approximately 60 sec. For imaging cAMP, islets were illuminated with a Lambda DG4 Wavelength Switcher (Sutter Instruments, Novato, CA) at 430 nm and two successive images were collected at 470 and 535 nm with a Cooke Sensican CCD camera. Data are expressed as the ratio of emission at 470 and 535 nm, F470/F535. For imaging Ca^2+^, islets were loaded with Fura2AM (4 µM), rinsed, and illuminated at 340 and 380 nm successively. Images were collected at 510 nm. Ca^2+^ imaging data are expressed as the ratio of excitation at 340 and 380 nm, F340/F380. We analyzed images with Imaging Workbench v5 software (Indec Biosystems, Santa Clara, CA).

### Glucose Tolerance Test

Five double transgenic Ins2-rtTA/pBI-cAMP male mice, 17–18 weeks old, were fasted overnight with water *ad libitum*. The mice were weighed, then injected i.p. with glucose (2 mg/g body weight). Plasma glucose levels were monitored using a One Touch Basic Glucometer on blood drawn at timed intervals from a tail vein. All five mice were then induced with two sequential dox injections at days 1 and 5 and were re-subjected to a glucose tolerance test on day 8.

## Supporting Information

Figure S1 Binary transgenic system for cAMP reporter mice. We used a cAMP reporter based on Green Fluorescent Protein variants (CFP and YFP), fused to Protein Kinase A subunits[1] and engineered this reporter into transgenic mice. Expression of the reporter is regulated in a cell-type directed and inducible manner, requiring crossing two separate strains of transgenic mice. In the Responder mouse (top right), the two subunits of the cAMP reporter (PKA-regulatory and PKA-catalytic) are fused at their C-termini to CFP and YPF, respectively. These chimeric cDNAs were inserted in opposite orientations into the pBI vector, flanking a bidirectional, tetracycline-inducible promoter. Transgenic mice with integrated copies of this construct (“pBI-cAMP” mice) do not express the reporter until induced with doxycycline (dox). In Transactivator mice (top left), reverse tetracycline transactivator (rtTA) is expressed either from a relatively non-selective promoter (CMV) or from a β-cell specific promoter (Ins2). We crossed pBI-cAMP mice (top right) separately with each of these two strains to produce double transgenic mice (bottom). In rtTA-expressing cells of such mice, cAMP reporter subunits are expressed in a dox-dependent fashion.
(0.34 MB TIF)

Figure S2 The cAMP reporter is insensitive to ΔpH in the physiologic range (pH 6.5 to 8.2). Because glucose causes transient increase and decrease of cytoplasmic pH in β-cells[1], [2], we considered whether glucose-evoked changes in fluorescence intensity might be attributable to any pH-sensitivity of our cAMP reporter subunits. We tested this in CHO cells transiently transfected with our enhanced cAMP reporter by deliberately alkalinizing, then acidifying the cytoplasm. Cells were first stimulated (grey bars) with fsk (20 µM) plus IBMX (100 µM). They were then treated with 20 mM NH_4_Cl in the recording buffer, which increases cytosolic pH, followed by acidification when NH_4_Cl is removed. This treatment produces pH changes spanning approx 1.7 pH units (i.e., pH 7.4 → pH 8.2 → pH 6.5), as reported by others[3]. This fluctuation of pH_i_ did not produce any discernible change in the FRET signal from the cAMP reporter (mean±s.e.m. for 8 cells in 1 experiment). This tested range of pH (⩆1.7 pH units) is considerably broader than occurs in β-cells upon glucose stimulation (<0.1 pH unit [1], [2]).
Another potential confound we considered is autofluorescence from NADH, produced in β-cells exposed to glucose[4], [5]. However, NADH fluorescence requires excitation below 400 nm and exhibits minimal emission at 535 nm. Consistent with this, we did not measure any glucose-stimulated changes in F470/F535 from wild-type islets or from transgenic islets that were not induced with dox (data not shown). Thus, neither cytoplasmic pH changes nor autofluorescent metabolites contaminate the cAMP-derived FRET ratio signals.
1. Juntti-Berggren L, Arkhammar P, Nilsson T, Rorsman P, Berggren PO (1991) Glucose-induced increase in cytoplasmic pH in pancreatic beta-cells is mediated by Na^+^/H^+^ exchange, an effect not dependent on protein kinase C. J Biol Chem 266: 23537-23541.
2. Stiernet P, Guiot Y, Gilon P, Henquin JC (2006) Glucose acutely decreases pH of secretory granules in mouse pancreatic islets. Mechanisms and influence on insulin secretion. J Biol Chem 281: 22142–22151.
3. Ozkan P, Mutharasan R (2002) A rapid method for measuring intracellular pH using BCECF-AM. Biochim Biophys Acta 1572: 143–148.
4. Dukes ID, McIntyre MS, Mertz RJ, Philipson LH, Roe MW, Spencer B, Worley JF, III (1994) Dependence on NADH produced during glycolysis for beta-cell glucose signaling. J Biol Chem 269: 10979–10982.
5. Rocheleau JV, Head WS, Piston DW (2004) Quantitative NAD(P)H/flavoprotein autofluorescence imaging reveals metabolic mechanisms of pancreatic islet pyruvate response. J Biol Chem 279: 31780–31787.
(0.49 MB TIF)

Table S1 This Table describes the yield and efficiency of the major steps during production of pBI-cAMP transgenic mice. We obtained three founder mice capable of expressing fluorescent reporter proteins in a dox-dependent fashion. Of the three lines of transgenic mice established, we extensively characterized one line (#5564) and used it in the present study.
(0.03 MB DOC)
