# Supplementary figures and images for: Imaging Cyclic AMP Changes in Pancreatic Islets of Transgenic Reporter Mice
Source: PLoS One. 2008 May 7;3(5):e2127. doi: 10.1371/journal.pone.0002127 (PMC2330161; doi:10.1371/journal.pone.0002127)

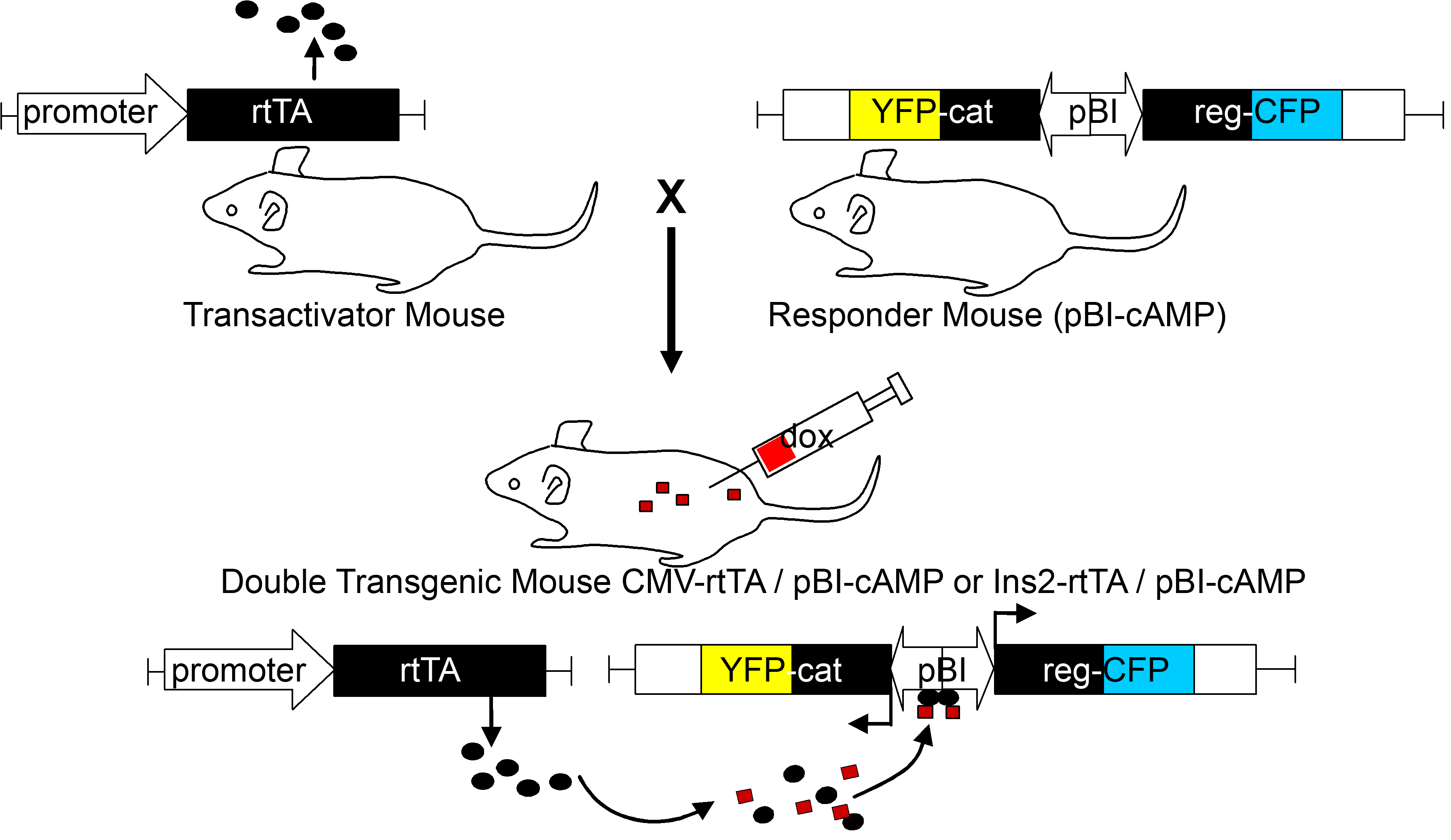

Supplement: Figure S1 — Binary transgenic system for cAMP reporter mice. We used a cAMP reporter based on Green Fluorescent Protein variants (CFP and YFP), fused to Protein Kinase A subunits[1] and engineered this reporter into transgenic mice. Expression of the reporter is regulated in a cell-type directed and inducible manner, requiring crossing two separate strains of transgenic mice. In the Responder mouse (top right), the two subunits of the cAMP reporter (PKA-regulatory and PKA-catalytic) are fused at their C-termini to CFP and YPF, respectively. These chimeric cDNAs were inserted in opposite orientations into the pBI vector, flanking a bidirectional, tetracycline-inducible promoter. Transgenic mice with integrated copies of this construct (“pBI-cAMP” mice) do not express the reporter until induced with doxycycline (dox). In Transactivator mice (top left), reverse tetracycline transactivator (rtTA) is expressed either from a relatively non-selective promoter (CMV) or from a β-cell specific promoter (Ins2). We crossed pBI-cAMP mice (top right) separately with each of these two strains to produce double transgenic mice (bottom). In rtTA-expressing cells of such mice, cAMP reporter subunits are expressed in a dox-dependent fashion. (0.34 MB TIF) [file pone.0002127.s001.tif]

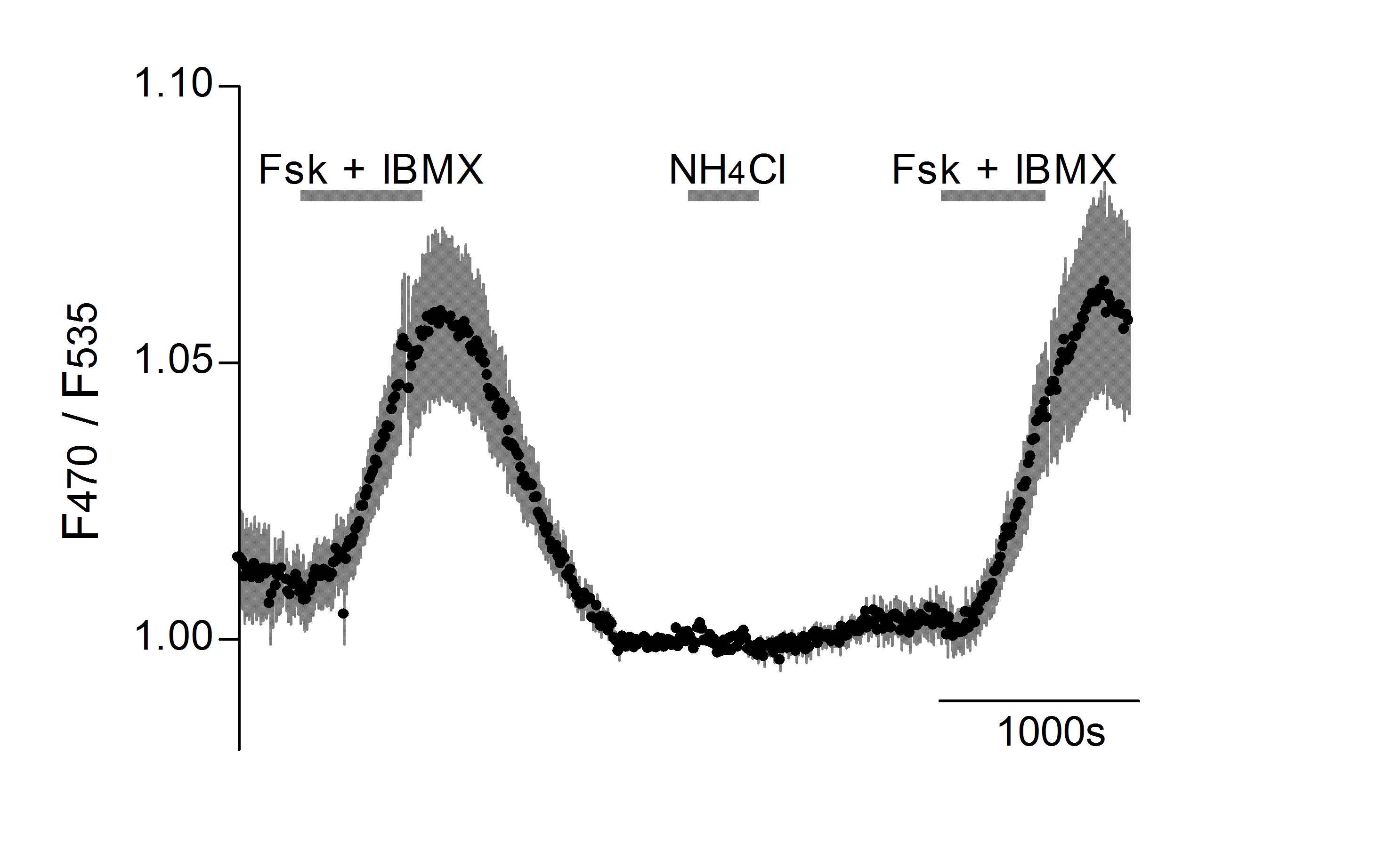

Supplement: Figure S2 — The cAMP reporter is insensitive to ΔpH in the physiologic range (pH 6.5 to 8.2). Because glucose causes transient increase and decrease of cytoplasmic pH in β-cells[1], [2], we considered whether glucose-evoked changes in fluorescence intensity might be attributable to any pH-sensitivity of our cAMP reporter subunits. We tested this in CHO cells transiently transfected with our enhanced cAMP reporter by deliberately alkalinizing, then acidifying the cytoplasm. Cells were first stimulated (grey bars) with fsk (20 µM) plus IBMX (100 µM). They were then treated with 20 mM NH4Cl in the recording buffer, which increases cytosolic pH, followed by acidification when NH4Cl is removed. This treatment produces pH changes spanning approx 1.7 pH units (i.e., pH 7.4 → pH 8.2 → pH 6.5), as reported by others[3]. This fluctuation of pHi did not produce any discernible change in the FRET signal from the cAMP reporter (mean±s.e.m. for 8 cells in 1 experiment). This tested range of pH (⩆1.7 pH units) is considerably broader than occurs in β-cells upon glucose stimulation (<0.1 pH unit [1], [2]). Another potential confound we considered is autofluorescence from NADH, produced in β-cells exposed to glucose[4], [5]. However, NADH fluorescence requires excitation below 400 nm and exhibits minimal emission at 535 nm. Consistent with this, we did not measure any glucose-stimulated changes in F470/F535 from wild-type islets or from transgenic islets that were not induced with dox (data not shown). Thus, neither cytoplasmic pH changes nor autofluorescent metabolites contaminate the cAMP-derived FRET ratio signals. 1. Juntti-Berggren L, Arkhammar P, Nilsson T, Rorsman P, Berggren PO (1991) Glucose-induced increase in cytoplasmic pH in pancreatic beta-cells is mediated by Na+/H+ exchange, an effect not dependent on protein kinase C. J Biol Chem 266: 23537-23541. 2. Stiernet P, Guiot Y, Gilon P, Henquin JC (2006) Glucose acutely decreases pH of secretory granules in mouse pancreatic islets. Me [file pone.0002127.s002.tif]
