# Supplementary material for: Imaging Cyclic AMP Changes in Pancreatic Islets of Transgenic Reporter Mice
Source: PLoS One. 2008 May 7;3(5):e2127. doi: 10.1371/journal.pone.0002127 (PMC2330161; doi:10.1371/journal.pone.0002127)
Supplement: Table S1 — This Table describes the yield and efficiency of the major steps during production of pBI-cAMP transgenic mice. We obtained three founder mice capable of expressing fluorescent reporter proteins in a dox-dependent fashion. Of the three lines of transgenic mice established, we extensively characterized one line (#5564) and used it in the present study. (0.03 MB DOC) [file pone.0002127.s003.doc]

**Supporting Table T1. Production of pBI-cAMP transgenic mice**

| # eggs injected | # recipient females | # successful pregnancies | # pups born | # transgenic mice | # mice expressing C-YFP and R-CFP |
| --- | --- | --- | --- | --- | --- |
| 143 | 7 | 6 | 34 | 7 | 3 |
